# Simultaneous treatment with sorafenib and glucose restriction inhibits hepatocellular carcinoma in vitro and in vivo by impairing SIAH1-mediated mitophagy

**DOI:** 10.1038/s12276-022-00878-x

**Published:** 2022-11-16

**Authors:** Jing Zhou, Ji Feng, Yong Wu, Hui-Qi Dai, Guang-Zhi Zhu, Pan-Hong Chen, Li-Ming Wang, Guang Lu, Xi-Wen Liao, Pei-Zhi Lu, Wen-Jing Su, Shing Chuan Hooi, Xin-Pin Ye, Han-Ming Shen, Tao Peng, Guo-Dong Lu

**Affiliations:** 1 Department of Physiology, School of Basic Medical Sciences, Guangxi Medical University, 530021 Nanning, Guangxi Province P. R. China; 2 Department of Physiology, National University of Singapore, Singapore, 117593 Singapore; 3 Department of Toxicology, School of Public Health, Guangxi Medical University, 530021 Nanning, Guangxi Province P. R. China; 4 Department of Hepatobiliary Surgery, First Affiliated Hospital of Guangxi Medical University, 530021 Nanning, Guangxi Province P. R. China; 5 Faculty of Health Sciences, University of Macau, Macau, P. R. China; 6 Key Laboratory of Early Prevention and Treatment for Regional High Frequency Tumor (Guangxi Medical University), Ministry of Education; Guangxi Key laboratory of High-Incidence-Tumor Prevention & Treatment (Guangxi Medical University), 530021 Nanning, Guangxi Province P. R. China

**Keywords:** Liver cancer, Mitophagy, Apoptosis

## Abstract

Transarterial chemoembolization (TACE) is the first-line treatment for unresectable intermediate-stage hepatocellular carcinoma (HCC). It is of high clinical significance to explore the synergistic effect of TACE with antiangiogenic inhibitors and the molecular mechanisms involved. This study determined that glucose, but not other analyzed nutrients, offered significant protection against cell death induced by sorafenib, as indicated by glucose deprivation sensitizing cells to sorafenib-induced cell death. Next, this synergistic effect was found to be specific to sorafenib, not to lenvatinib or the chemotherapeutic drugs cisplatin and doxorubicin. Mechanistically, sorafenib-induced mitophagy, as indicated by PINK1 accumulation, increased the phospho-poly-ubiquitination modification, accelerated mitochondrial membrane protein and mitochondrial DNA degradation, and increased the amount of mitochondrion-localized mKeima-Red engulfed by lysosomes. Among several E3 ubiquitin ligases tested, SIAH1 was found to be essential for inducing mitophagy; that is, SIAH1 silencing markedly repressed mitophagy and sensitized cells to sorafenib-induced death. Notably, the combined treatment of glucose restriction and sorafenib abolished ATP generation and mitophagy, which led to a high cell death rate. Oligomycin and antimycin, inhibitors of electron transport chain complexes, mimicked the synergistic effect of sorafenib with glucose restriction to promote cell death mediated via mitophagy inhibition. Finally, inhibition of the glucose transporter by canagliflozin (a clinically available drug used for type-II diabetes) effectively synergized with sorafenib to induce HCC cell death in vitro and to inhibit xenograft tumor growth in vivo. This study demonstrates that simultaneous treatment with sorafenib and glucose restriction is an effective approach to treat HCC, suggesting a promising combination strategy such as transarterial sorafenib-embolization (TASE) for the treatment of unresectable HCC.

## Liver cancer: Restricting glucose helps chemotherapy to work

A new approach to treating the most common type of liver cancer, hepatocellular carcinoma, combines the chemotherapy drug sorafenib with procedures that restrict the supply of glucose to the diseased tissues. Jing Zhou at Guangxi Medical University, Nanning, China, and coworkers investigated the effects of this strategy in cultured cells and on mice with tumors that modeled liver cancers. They confirmed that glucose protects cells against the cancer cell-killing properties of sorafenib, but this could be effectively counteracted by restricting glucose supply. The drug and glucose-restriction combination almost halted tumor growth in mice. Restricting other nutrients did not show the same effect, revealing a specific role for glucose and suggesting possible combination treatments of sorafenib and embolization. The researchers also identified key aspects of the molecular mechanism behind the combined effect.

## Introduction

Hepatocellular carcinoma (HCC) ranks as the third leading cause of cancer death worldwide^1^. Because of late diagnosis, most patients present with unresectable intermediate stage HCC (BCLC-B), for which the median overall survival (OS) is 16 months, or advanced-stage HCC (BCLC-C), for which the OS is 6–8 months^2^. Transarterial chemoembolization (TACE) is the standard treatment for intermediate HCC. This surgery involves embolization with intra-arterial infusion of chemotherapeutic agents (single or combination regimens of doxorubicin, epirubicin, cisplatin, and miriplatin) to achieve both ischemic and chemocytotoxic effects^2^. Notably, several studies have revealed that chemotherapeutic agents may be dispensable^3–5^; specifically, no differences in tumor response or OS was observed in TACE-treated HCC patients and patients who went transarterial embolization (TAE) without chemotherapeutic drugs. A recent systemic review pooled the data from 10,108 HCC patients in 101 studies and determined that the median OS of TACE-treated patients was 19.4 months^6^. With the revolutionary introduction of immune checkpoint inhibitors for the management of advanced HCC, there is still a large demand to increase the efficacy of TACE for patients with intermediate HCC.

Embolization-induced ischemia causes both hypoxia and metabolic stress. Hypoxia leads to increases in hypoxia-inducible factor-1*α* (HIF1A) and stimulates vascular endothelial growth factor (VEGF)-mediated angiogenesis, which in turn promotes cancer recurrence^7^. It is thus tempting to postulate that the antiangiogenic drugs sorafenib and lenvatinib, which target multiple kinases, including VEGF receptors, and serve as first-line treatments for advanced HCC^8,9^, can synergize with TACE to prolong patient survival. Sequential treatments of sorafenib and TACE showed promising results in a few Phase II and Phase III randomized clinical trials^10–13^. Our pooled-data meta-analysis also showed that the combination of TACE and sorafenib treatment was beneficial (HR = 0.81, 95% CI 0.71–0.94, Supplementary Fig. 1a). However, a deeper mechanical understanding of the interplay between TACE and sorafenib is needed to design more effective and safer combination strategies.

Similar to the proangiogenic action of hypoxia, metabolic stress may activate autophagic mechanisms and subsequent utilization of intracellular/extracellular nutrient fuels may meet cell survival needs^14,15^. For example, studies performed in our laboratory and those performed by others revealed that starved cancer cells catabolized cellular and microenvironmental lipids through lipophagy (a selective form of autophagy in which lipids are digested) to drive energy metabolism^16–18^. Other nutrient fuels utilized by starved cancer cells include acetate, ketone, lactate, and branched amino acids^14–16^. Hence, adaptive cancer cells escape cell death during the acute phase of embolization and then proliferate and invade new metastatic sites. Consistently, inhibition of autophagy using either chloroquine or Lys05 potentiated the anticancer effect of TACE in preclinical rat and rabbit models, respectively^19,20^.

Mitochondria are both powerhouses of energy generation and central metabolic hubs for nucleotide, fatty acid, and amino acid substrates. To preserve and restore essential mitochondrial homeostasis, mitochondrial quality control mechanisms have evolved through endosymbiosis^21^; however, mitochondrial impairment may result in cell and tissue damage. Mitophagy, a mitochondrion-selective form of autophagy, is a mechanism through which a portion or an entire mitochondrion is eliminated under physiological or pathological conditions^22^. The mitophagy regulatory pathway is best exemplified by the PINK1 (phosphatase and tensin homolog induced kinase 1)-PRKN (Parkin RBR E3 ubiquitin protein ligase) axis^23,24^. PINK1, which is activated by autophosphorylation and stabilized upon mitochondrial injury, can recruit and activate PRKN at the mitochondrial surface^25^. These two proteins coordinate to generate phosphorylated polyubiquitin (phospho-poly-Ub) chains. Functioning as ‘eat-me’ signals, the resulting phospho-poly-Ub chains are recognized by autophagy adapters. Recent studies showed that mitophagy can be independent of PRKN^26^.

In the present study, we determined the combined effect of sorafenib and glucose deprivation on HCC cell death in vitro and in vivo. Furthermore, the involvement of mitophagy and mitochondrial dysfunction was determined.

## Materials and methods

### Chemicals

Sorafenib (#T0093L) was purchased from TargetMol (Shanghai, China); lenvatinib (#S1164), brivanib (#S1084) and bafilomycin A1 (#S1413) were purchased from Selleck (Shanghai, China); cisplatin (#P4394), doxorubicin (#D1515), *N*-acetyl-l-cysteine (NAC, #A7250), and Hoechst (#94403) were purchased from Sigma‒Aldrich (Shanghai, China); canagliflozin (#A11100) was purchased from AdooQ Bioscience (Nanjing, China); and oligomycin (#HY-N6782), antimycin A (#HY-105755), phloretin (#HY-N0142), z-VAD-FMK (#HY-16658B), necrostatin-1 (#HY-15760), and ferrostatin-1 (#HY-100579) were purchased from MedChemExpress (Shanghai, China).

### Cell culture, treatment, and siRNA silencing

Most cell lines were obtained from the American Type Culture Collection and cultured in either DMEM or RPMI 1640 medium with 10% FBS (Gibco, Newcastle, Australia) and 100 U/ml penicillin/streptomycin in a 5% CO_2_ incubator. HCC-M cells^27^ were kindly provided by Dr E.C. Ren (National University of Singapore). Nutrient levels in cell medium were restricted as previously described^17^. After brief rinsing with PBS twice, the cells were starved in Earle’s balanced salt solution (with 1 g/L glucose) or dual glucose-free and glutamine-free DMEM (Thermo-Fisher). Cell death and survival were measured by subG1, propidium iodide (PI, Sigma‒Aldrich) exclusion assay or lactate dehydrogenase (LDH) assays^17^. Transient silencing was performed using Lipofectamine RNAiMAX transfection reagent with siRNAs specific for several known E3 ubiquitin ligases involved in mitophagy (PINK1, SIAH1, STUB1, and MUL1), all obtained from Thermo-Fisher.

### Measurement of ATP and mitochondrial respiratory oxygen consumption rates

The ATP concentrations were determined by assay kits obtained from BioVision (#K354, Milpitas, USA), and mitochondrial oxygen consumption rates (OCRs) were determined with a Seahorse XF24 analyzer obtained from Agilent (North Billerica, USA) as described previously^17^. Briefly, after treatment with sorafenib in XF medium with/without glucose for 1 h, the OCRs were determined on the basis of the manufacturer’s protocol.

### Immunofluorescence and time-lapse microscopy

Mitochondrial DNA (mtDNA; #61014) was visualized on an Olympus FV3000 Confocal Laser Scanning Microscope as previously described^28^. Ten images containing more than 300 cells from three independent experiments were analyzed with Imaris 9.1 software. mKeima-Cox8-treated HeLa cells were prepared in Dr. HM Shen’s laboratory.

### Western blot analysis

Western blotting was performed as described previously (19). Primary antibodies against PINK1 (#6946), PRKN (#4211), TOMM20 (#42406), COX4I1/Cox IV (#4850), MFN1 (#14739), MFN2 (#9482), STUB1/CHIP (#2080 S), SMURF1 (#2174), phospho-ubiquitin (Ser65, #62802), and MAP1LC3B/LC3B (#3868) were purchased from Cell Signaling (Beverly, MA, USA); SQSTM1/p62 (#sc-48402), ACTB (#sc-47778), and SLC5A2/SGLT2 (#sc-393350) from Santa Cruz Biotechnology (Dallas, TX, USA); phospho-ubiquitin (Ser65, ABS1513-I) from Merck; TIMM23 (#611223) from BD Biosciences; MTCO2/Cox II (#ab110258), MUL1 (#ab209263), and SIAH1 (#ab2237) from Abcam (Shanghai, China).

### 3D spheroid tumor models

Tumor cell 3D spheroids were generated by plating HepG2 or Huh7 cells into 96-well U-shaped-bottom Nunclon Sphera microplates (#174925, Thermo-Fisher)^29^. After the formation of a single spheroid, cells were treated with 5 μM sorafenib with/without 20 μM canagliflozin. Cell viability was analyzed using a Calcein/PI Cell Viability/Cytotoxicity Assay Kit (#C2015M, Beyotime, Shanghai, China). Fluorescent images of calcein (green)-/PI (red)-stained spheroids were obtained using an EVOS™ FL Auto Imaging System (Thermo).

### HCC xenograft experiment

The HCC xenograft experiment was approved by the Guangxi Medical University Institutional Animal Care and Use Committees (#201910029) and performed according to the Association for Assessment and Accreditation of Laboratory Animal Care guidelines. A total of 32 male BALB/c nude mice were purchased from Hunan SJA Laboratory Animal Co., Ltd. (Changsha, China) and housed in an SPF laboratory with free access to food and water. Five million Huh7 cells were inoculated subcutaneously into the right flanks of nude mice. Once the inoculated tumor size reached 40–60 mm^3^, the mice were randomly separated into 4 groups (*N* = 8 per group, 4 per cage). The mice were then treated with sorafenib alone (20 mg/kg mouse weight), canagliflozin alone (30 mg/kg), the combination treatment (20 mg/kg sorafenib plus 30 mg/kg canagliflozin), or control PBS by gavage once every other day. Tumor size was measured by caliper daily and calculated using the formula (length × width^2)/2. All mice were killed on the 15th day of treatment, and tumor nodules were collected for pathological examination and other assays. The IHC experiment was carried out as described previously^17^.

### Statistical analyses

The experimental differences were assessed by SPSS 22.0 software via ANOVA LSD or two-sided Student’s *t* test. Statistical significance was defined as a P value <0.05.

For details on the other methods used in this study, please refer to the supplementary material and methods.

## Results

### Glucose offered significant protection against sorafenib-induced cell death

**Fig. 1:**
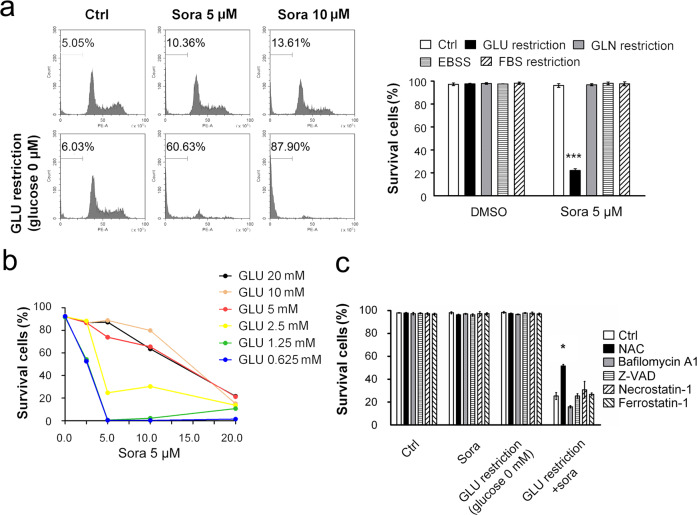
Glucose restriction sensitized HCC cells to sorafenib-induced cell death in vitro. **a** Huh7 cells were treated with different types of nutrient-deficient media in the presence or absence of sorafenib (Sora) for 8 h. The proportion of dead cells was measured by both subG1 cell cycle (left panel) and PI exclusion assays (right). **b** Huh7 cells were incubated in DMEM containing various concentrations of glucose (as indicated) with sorafenib. **c** Huh7 cells were pretreated with NAC (5 mM), bafilomycin A1 (50 nM), z-VAD (40 μM), necrostatin-1 (30 μM) or ferrostatin-1 (5 μM) for 30 min before sorafenib (5 μM) treatment in glucose-free medium for another 8 h. *P < 0.05; ***P < 0.001.

Embolization causes nutrient inaccessibility of glucose, growth factors, glutamine, and other amino acids, as well as hypoxia. To determine the specific nutrient that was essential for HCC cell survival after sorafenib treatment, we treated HCC cells with sorafenib and cultured them in different types of restriction media, including glucose (GLU)-free DMEM, glutamine (GLN)-free DMEM, amino acid-free EBSS and FBS-free DMEM. Glucose restriction, but not restriction of the other nutrients, synergized with sorafenib to cause cell death (Fig. 1a). Adding glucose to the glucose-free medium rescued cells from cell death in a dose-dependent manner (Fig. 1b). The synergistic effects were reproducible with HepG2, Huh7, and HCC-M cell lines, with results evident within 8 h of treatment; this effect was validated via both subG1 (Supplementary Fig. 1b) and LDH release assays (Supplementary Fig. 1c). Furthermore, the sensitization of cells to death was independent of necrosis, apoptosis, and ferroptosis, as indicate by pretreatment with the respective inhibitors necrostatin-1, z-VAD or ferrostatin-1 failing to change the cell death rate (Fig. 1c). In contrast, NAC (a ROS scavenger) partially prevented cell death. Taken together, these results suggest that a synergistic effect was realized with sorafenib in conjunction with glucose restriction to induce HCC cell death.

### Sorafenib and glucose restriction synergistically induced cell death

**Fig. 2:**
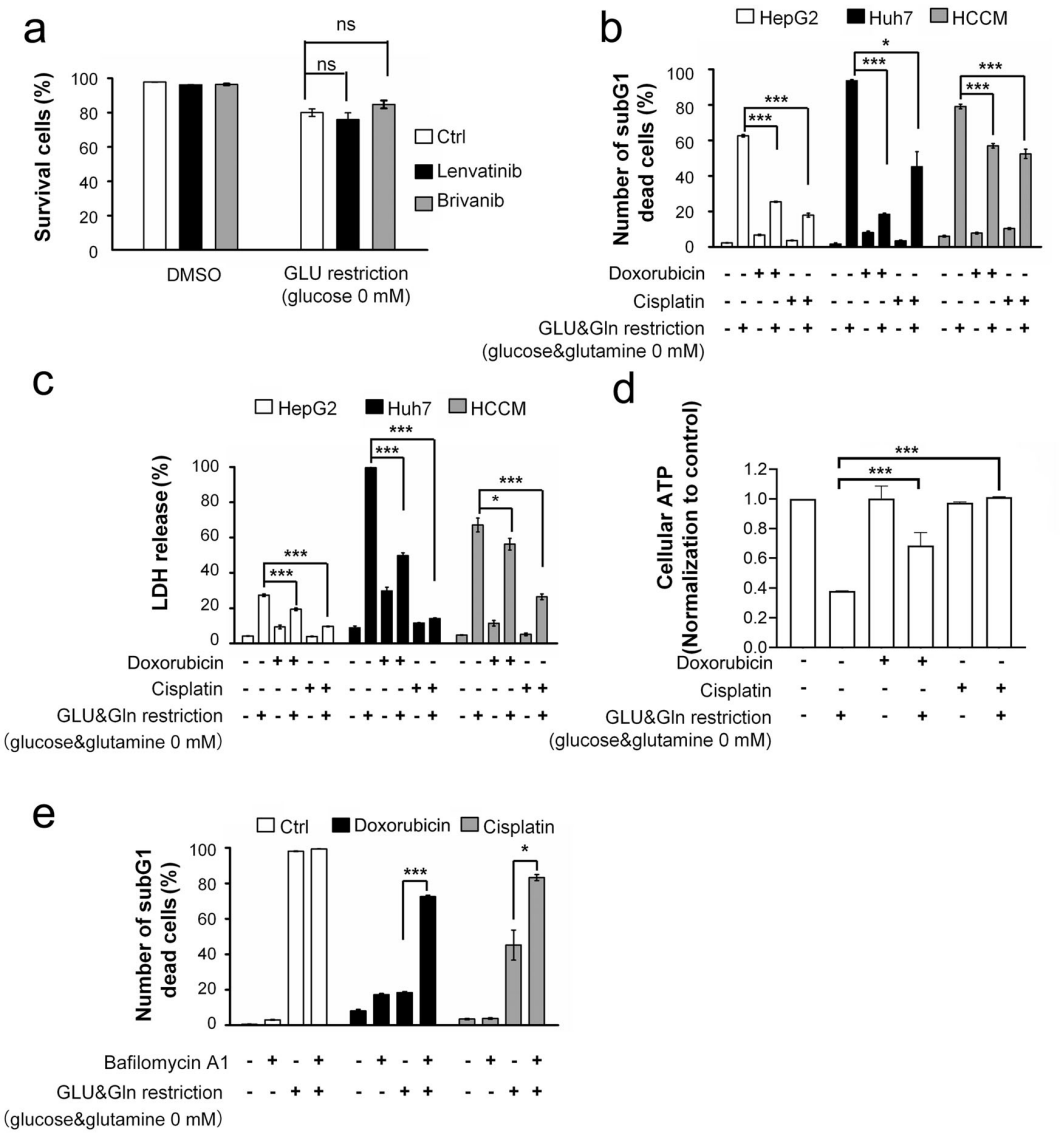
Sorafenib specifically synergized with glucose restriction to induce cell death. **a** Huh7 cells were treated with 10 μM lenvatinib or brivanib with or without glucose. ns, no significant difference. **b**, **c** HCC cells were treated with cisplatin (220 μM) or doxorubicin (2 μg/ml) in the glucose-free medium. Cell death was determined by subG1 (**b**) and LDH release assays (**c**). **d** Huh7 cells were treated with cisplatin (220 μM) or doxorubicin (2 μg/ml) in glucose-free medium before detection of cellular ATP contents. **e** Huh7 cells were pretreated with bafilomycin A1 (50 nM) for 30 min before treatment with cisplatin (220 μM) or doxorubicin (2 μg/ml) in the glucose-free medium. *P < 0.05; ***P < 0.001.

Next, we explored whether the cell death is induced by other HCC clinical treatments. Neither lenvatinib (another first-line antiangiogenic drug used for advanced HCC) nor brivanib (a multitargeted tyrosine kinase inhibitor) showed an additive effect on the death of cells under glucose-restricted conditions (Fig. 2a), which was a different outcome when sorafenib was used (Fig. 1). Furthermore, cisplatin and doxorubicin, which are frequently applied via TACE treatment, exhibited the opposite effects by protecting different HCC cell lines from death induced by long-term (1–2 days) glucose restriction (Fig. 2b). The protective effects were confirmed by LDH release assay (Fig. 2c) and ATP content assay (Fig. 2d). Given that energy depletion triggers macroautophagy^30^, we tested the role of macroautophagy by adding a lysosome inhibitor to the culture. Pretreatment with the lysosome inhibitor bafilomycin A1 abolished cisplatin- and doxorubicin-mediated protection (Fig. 2e), suggesting the possible involvement of the autophagy‒lysosome pathway. Therefore, sorafenib, but not other commonly used HCC drugs, was specific in its synergistic effect with glucose restriction.

### The combination treatment impaired mitochondrial function

**Fig. 3:**
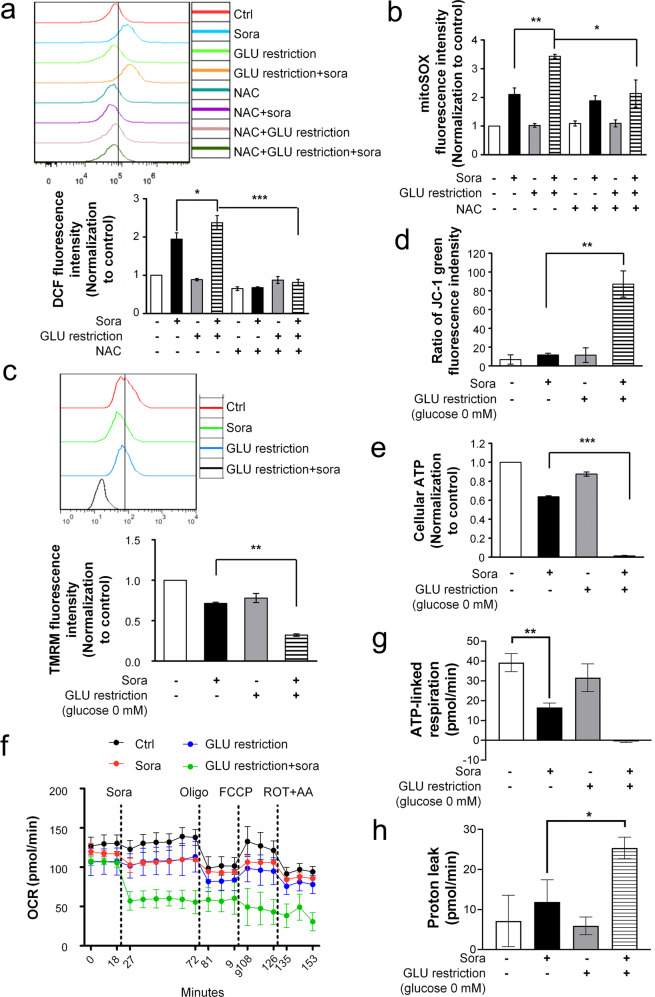
The combination of sorafenib and glucose restriction impaired mitochondrial functions. **a** After the indicated treatments, ROS levels were determined by DCF assay, and the summarized data is shown in the bottom panel of **a**. *P < 0.05; ***P < 0.001. **b** Mitochondrion-derived ROS were measured by the MitoSOX Red assay. *P < 0.05; **P < 0.01. **c**, **d** The mitochondrial membrane potential was determined by TMRM (**c**) or JC-1 assay (**d**) after the indicated treatments. **e** The cellular ATP levels were determined after treatment with sorafenib in the glucose-free medium for 2 h. **f** Mitochondrial oxygen consumption rates after sequential treatments with sorafenib in the presence or absence of glucose restriction medium for 1 h and then with individual mitochondrial poisons (*N* = 3 or 4) were measured by Seahorse mitochondrial stress analysis. Oligo oligomycin, FCCP carbonyl cyanide-4 (trifluoromethoxy) phenylhydrazone, Rot rotenone, AA antimycin A. **g**, **h** The OCRs associated with ATP generation (**g**) and proton leakage (**h**) were calculated based on the results shown in **f**. *P < 0.05; **P < 0.01.

We then investigated whether mitochondria are critical to the synergistic effect because of the multiple important roles they play in energy metabolism, ROS production and cell death. First, sorafenib alone caused enhanced production of general ROS (Fig. 3a, showing DCF assay results) and mitochondria-derived ROS (Fig. 3b, showing MitoSOX results). The combination of sorafenib and glucose restriction increased their production. NAC prevented these redox changes, which is in line with its prosurvival effects (Fig. 1c). Second, the combination treatment, but not sorafenib alone or glucose restriction alone, disrupted the mitochondrial membrane potential (MMP), as evidenced by a decrease in TMRM mitochondrial retention (Fig. 3c) and low levels of fluorescent JC-1 transition (Fig. 3d). More importantly, sorafenib and glucose restriction synergistically abolished ATP generation (Fig. 3e) and ATP-associated respiratory OCR (Fig. 3f, g, showing Seahorse metabolic assay results), while sorafenib alone exhibited moderate inhibitory effects. Furthermore, the combined treatment significantly enhanced proton leakage-associated OCR (Fig. 3h), which is consistent with MMP impairment (Fig. 3c, d). In contrast, neither lenvatinib nor brivanib reproduced the inhibitory effects on mitochondrial OCR (Supplementary Fig. 2a, b).

### The combination treatment abolished sorafenib-induced mitophagy

**Fig. 4:**
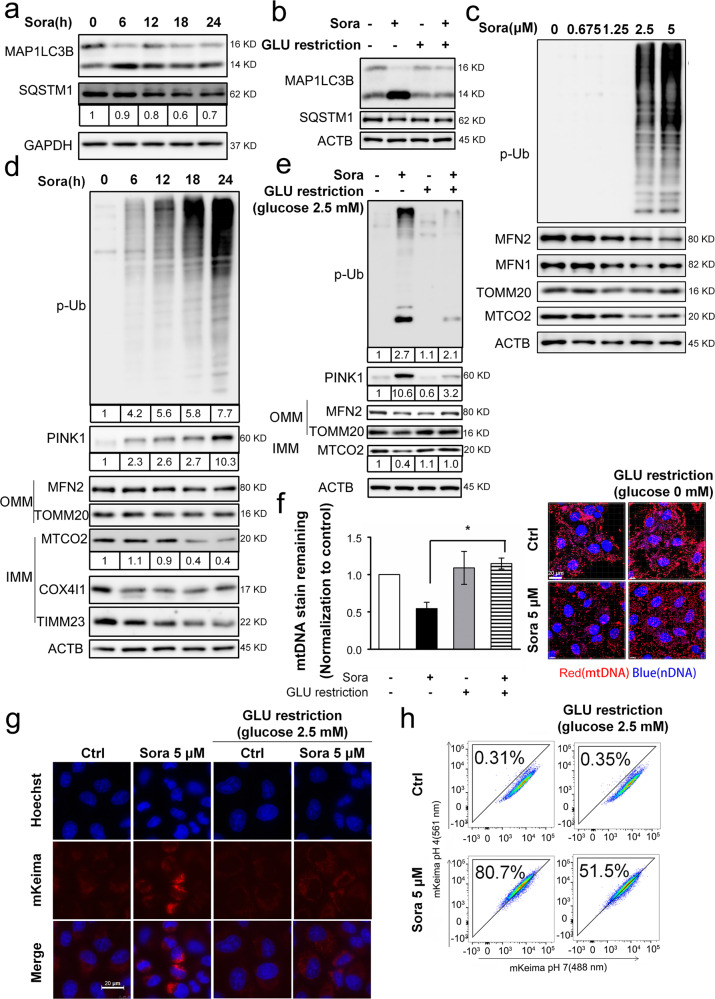
The combination treatment abolished sorafenib-induced mitophagy. **a** Huh7 cells were treated with sorafenib (5 μM) over a time course, and then, the protein levels were measured by Western blotting. The protein abundances were determined with ImageJ software, and the values are listed below the blots. **b** Huh7 cells were treated with sorafenib (5 μM) in glucose-free medium for 4 h. **c**, **d** Huh7 cells were treated with sorafenib (5 μM) in a dose- (**c**) or time-dependent manner (**d**). **e** The cells were treated as described in **b**. **f** The mitochondrial DNA (mtDNA) was imaged, and representative images are shown in the right panel, while the fluorescence intensities were analyzed by Imaris 9.1 software, and the results are summarized in the left panel. *P < 0.05. **g**, **h** HeLa cells with stable mKeima-parkin expression were treated with sorafenib (5 μM) in glucose-free medium for 6 h. The change in fluorescence intensity after engulfment of mKeima-parkin in lysosomes was detected by confocal microscopy (**g**) or flow cytometry (**h**).

We then examined the possible involvement of mitophagy in the synergistic effect of sorafenib and glucose restriction. By recycling impaired or excessive mitochondria, mitophagy restricts the leakage of mitochondrial proteins and redox species^21,22^. Sorafenib alone caused macroautophagy, as evidenced by the typical macroautophagic parameters, such as MAP1LC3B/LC3 conversion and SQSTM1/p62 degradation (Fig. 4a). However, these changes were abolished when sorafenib was combined with glucose restriction (Fig. 4b). More importantly, sorafenib promoted changes characteristic of mitophagy: (1) dose- and time-dependent protein modifications with phospho-poly-Ub chain (Fig. 4c, d); (2) decreased protein abundance on both the outer mitochondrial membrane (OMM, such as MFN2 and TOMM20) and inner mitochondrial membrane (IMM, such as MTCO2/Cox II, COX4I1/Cox IV, and TIMM23; Fig. 4c, d); (3) decreased mtDNA contents (Fig. 4f); and (4) increased engulfment of mitochondria-localized mKeima-Red lysosomes, which are acidic organelles (Fig. 4g, h). These mitophagic changes were decreased or even abolished when sorafenib was combined with glucose restriction (Fig. 4e–g). Bafilomycin A1 pretreatment sensitized cells to sorafenib-induced cell death (data not shown), suggesting the involvement of autophagy/mitophagy.

### Mitochondrial energy disruption abolished mitophagy

**Fig. 5:**
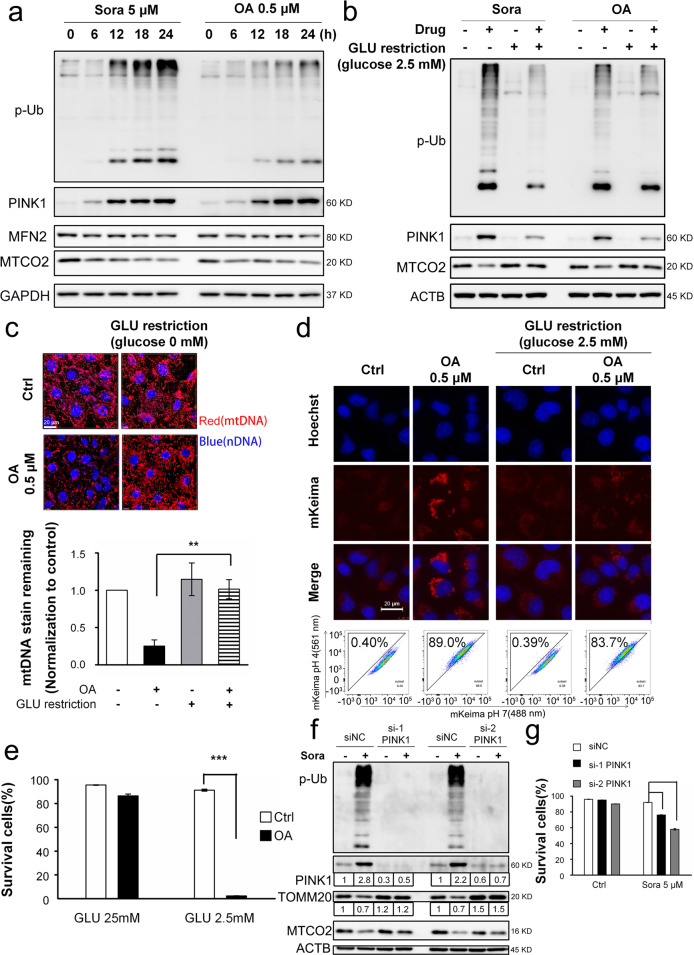
Mitochondrial energy disruption abolished PINK1-mediated mitophagy. **a** Huh7 cells were treated with oligomycin/antimycin (OA, 0.5 μM for both) or sorafenib (5 μM) over a time course. **b** Huh7 cells were treated with OA (0.5 μM) or sorafenib (5 μM) in glucose-free medium. **c** After cells were treated with OA for 2 h, mtDNA was measured, and the results are shown in Fig. 4f. **P < 0.01. **d** The change in the fluorescence of mKeima-parkin was measured, and the results are shown in Fig. 4g and h. **e** Huh7 cells were treated with OA (0.5 μM) in glucose-free medium for 24 h. Cell death was determined by PI exclusion assay as shown in Fig. 1. ***P < 0.001. **f**, **g** Huh7 cells were transfected with siRNA targeting PINK1 before treatment with sorafenib (5 μM) for 24 h, and protein abundance was measured by western blotting (**f**). Cell death (**g**) was analyzed, and the results are shown in **e**. ^#^*P* < 0.05; ^###^*P* < 0.001.

We postulated that the synergistic effects on mitophagy and cell death may result from mitochondrial failure to maintain ATP generation because glucose restriction represses glycolysis, while sorafenib directly inhibits the activity of mitochondrial electron transport chain (ETC) complex enzymes^31^, two of which are essential energy-generating pathways. Oligomycin and antimycin (OA) are two extensively studied mitochondrial poisons targeting ETC complexes and are capable of activating mitophagy^32^. We then examined whether OA exerts an effect similar to that of sorafenib to produce a synergistic effect with glucose restriction. OA-induced mitophagy, as indicated by time-dependent phospho-poly-Ub chain modification, a reduction in MFN2 and MTCO2/COX II protein levels (Fig. 5a, b), degradation of mtDNA (Fig. 5c) and engulfment of mitochondria by lysosomes (Fig. 5d). As expected, the combined treatment of glucose restriction and OA reversed these changes. More importantly, glucose restriction sensitized cells to OA-induced cell death (Fig. 5e).

We noted that the marked increase in the degree of phospho-poly-Ub modification was accompanied by PINK1 upregulation after sorafenib (Fig. 4d) and OA treatment (Fig. 5a). In contrast, the respective drug treatments with glucose restriction abolished these changes (Figs. 4e and 5b). PINK1 is an essential kinase for ubiquitin phosphorylation in mitophagy^25^. Notably, silencing PINK1 via specific siRNAs abolished sorafenib-induced mitophagy-related protein level changes (Fig. 5f), validating the essential role played by PINK1 in mitophagy induction^32^. PINK1 silencing further sensitized sorafenib to cell death even under glucose-replete conditions (Fig. 5g). Therefore, PINK1 may be, at least partially, critical to sorafenib-induced mitophagy.

### SIAH1 played an essential role in sorafenib-induced mitophagy

**Fig. 6:**
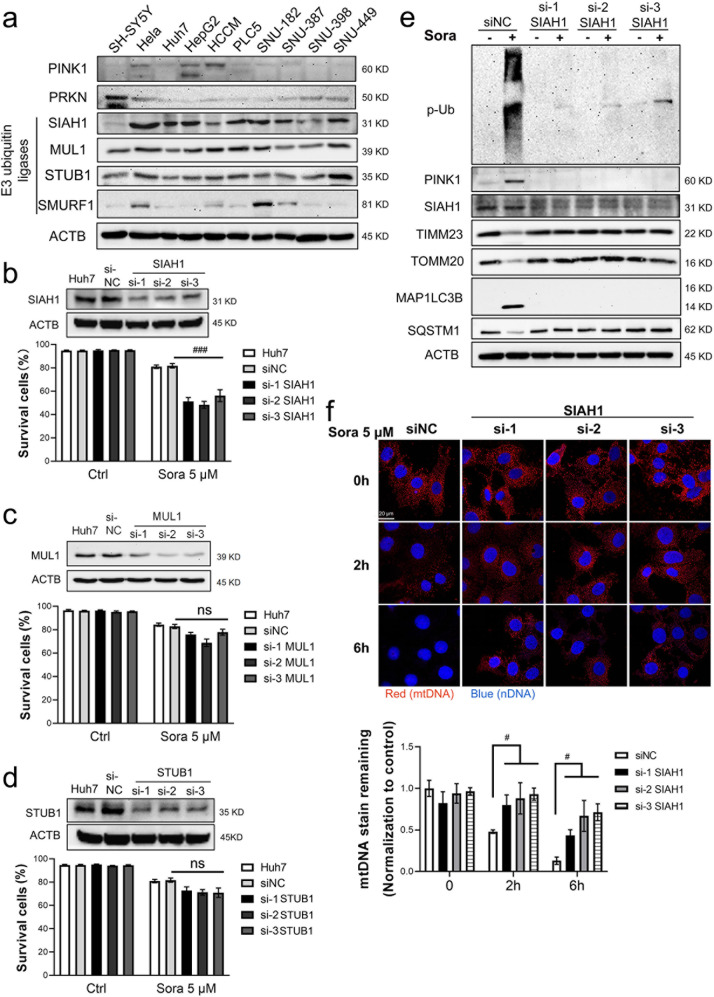
SIAH1 played an essential role in PINK1-mediated mitophagy. **a** PRKN protein levels in different HCC cells were analyzed by Western blotting. SH-SY5Y cells were used as the positive controls for PRKN expression. **b**–**d** Huh7 cells were transfected with siRNA targeting SIAH1 (**b**), MUL1 (**c**), or STUB1 (**d**) before treatment with sorafenib (5 μM) for 24 h. ^###^*P* < 0.001. **e** SIAH1-silenced Huh7 cells were treated with sorafenib (5 μM) for 20 h, and then, the protein levels were measured by western blotting. **f** After treatment with sorafenib for the indicated times, mtDNA was measured, and the results are shown in Fig. 4f, and the fluorescence intensities were analyzed, and the results are summarized in the lower panel, ^#^*P* < 0.05.

The PINK1-PRKN axis is the most extensively studied axis involved in mitophagy^23^. However, the PRKN level was decreased or depleted in 50% of primary HCC tissues^33^. Similarly, the PRKN level was low or undetectable in most tested HCC cell lines (Fig. 6a). Hence, we speculated that other E3 ubiquitin ligases, e.g., SIAH1^34^, MUL1^35^, STUB1^36^, and SMURF1^37^, which have been reported to participate in mitophagy, were involved in sorafenib-induced mitophagy. SIAH1, MUL1, and STUB1 were detectable in all the tested HCC cell lines, while SMURF1 was absent in more than 50% of the HCC cell lines, including the HepG2 and Huh7 cell lines (Fig. 6a). More importantly, silencing SIAH1 (Fig. 6b), but not MUL1 (Fig. 6c), or STUB1 (Fig. 6d), abolished sorafenib-induced mitophagy and sensitized cells in glucose-replete medium to death. In particular, knocking down SIAH1 expression abolished sorafenib-induced phospho-poly-Ub chain accumulation, PINK1 stabilization, mitochondrial protein degradation (TIMM23 and TOMM20, Fig. 6e), and mtDNA loss (Fig. 6f). SIAH1 silencing (but that of not MUL1 or STUB1) also repressed the changes in LC3 conversion and p62 degradation (Fig. 6e and Supplementary Fig. 3). Taken together, these data indicated that SIAH1 might function as a major E3 ligase to activate sorafenib-induced mitophagy.

### Sorafenib and pharmacological inhibition of glucose transporters showed a synergistic effect

**Fig. 7:**
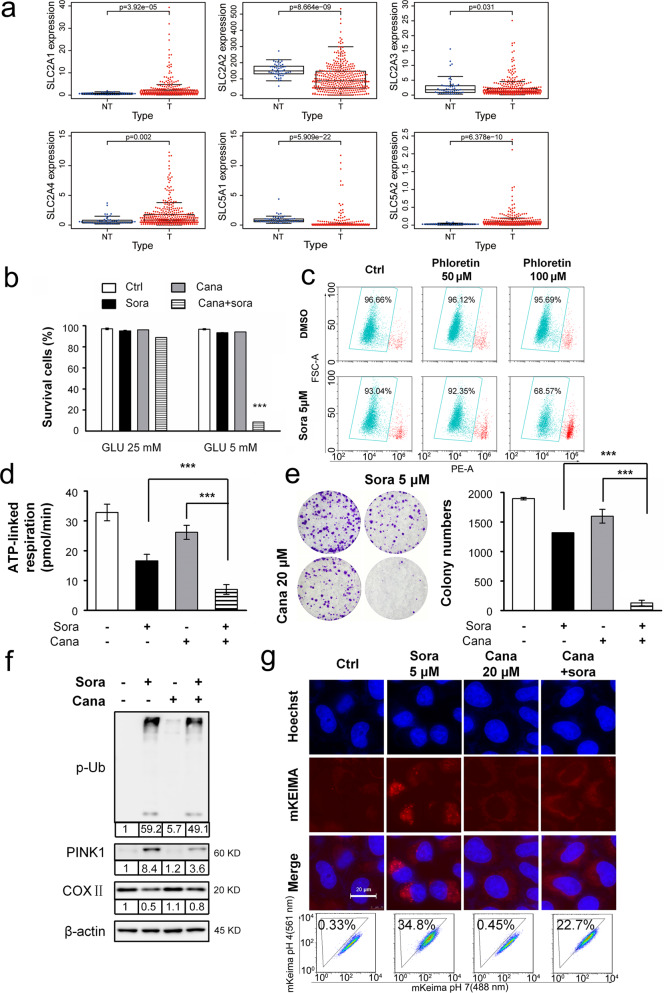
Inhibition of glucose transporters sensitized cells to sorafenib- induced death. **a** Expression of the respective glucose transporter between HCC tissues (*n* = 374) and adjacent noncancerous tissues (*n* = 50). **b** Huh7 cells were treated with sorafenib (5 μM) and/or canagliflozin (cana, 20 μM) in media with high (25 mM) and low (5 mM) levels of glucose for 24 h. ***P < 0.001. **c** Huh7 cells were treated with sorafenib (5 μM) and/or phloretin (50 and 100 μM) for 24 h. **d** The mitochondrial OCR associated with ATP generation was determined as described in Fig. 3g in cells treated with a combination of sorafenib and canagliflozin. **e** Huh7 cells were treated with sorafenib (5 μM) and canagliflozin (20 μM) for 6 h. Then, the medium was replaced with fresh medium, and the cells were cultured for an additional 10 days. **f** Huh7 cells were treated with sorafenib and canagliflozin for 20 h. **g** The mKeima-parkin fluorescence was determined as described in Fig. 4g after the designated treatments.

Glucose restriction can be achieved by inhibition of the glucose transporter, another clinically feasible method to mimic glucose deprivation in vitro. Exploration into common glucose transporters using TCGA data revealed that SLC2A1 (glucose transporter 1) and SLC5A2 (sodium/glucose cotransporter 2) were significantly overexpressed in HCC tissues (*P* < 0.001) compared to normal liver tissues (Fig. 7a and Supplementary Fig. 4a, b). The tested HCC cell lines overexpressed at least one of these two proteins (Supplementary Fig. 4c). We thus examined the effect of the inhibitors canagliflozin against SLC5A2^38^ and phloretin against SLC2A1^39^. Canagliflozin (20 μM) decreased glucose transportation (Supplementary Fig. 4d) and, more importantly, synergized with sorafenib to induce cell death under glucose-replete conditions (Fig. 7b). Phloretin exhibited a similar effect but only at the high dose of 100 μM (Fig. 7c), which might not be achievable in vivo. Therefore, we chose canagliflozin for further investigation. The canagliflozin and sorafenib combination treatment inhibited ATP generation, reduced mitochondrial OCR (Fig. 7d) and long-term colony growth (Fig. 7e). In addition, canagliflozin repressed multiple sorafenib-induced mitophagy changes, including PINK1 upregulation, phospho-poly-Ub chain modification, mitochondrial protein MTCO2 degradation (Fig. 7f) and lysosomal engulfment of mitochondrion-localized mKeima-Red (Fig. 7g). Taken together, these data suggested that simultaneous treatments with canagliflozin abolished sorafenib-induced mitophagy and sensitized cells to death.

### Combined treatment with canagliflozin and sorafenib inhibited HCC xenograft tumor growth

**Fig. 8:**
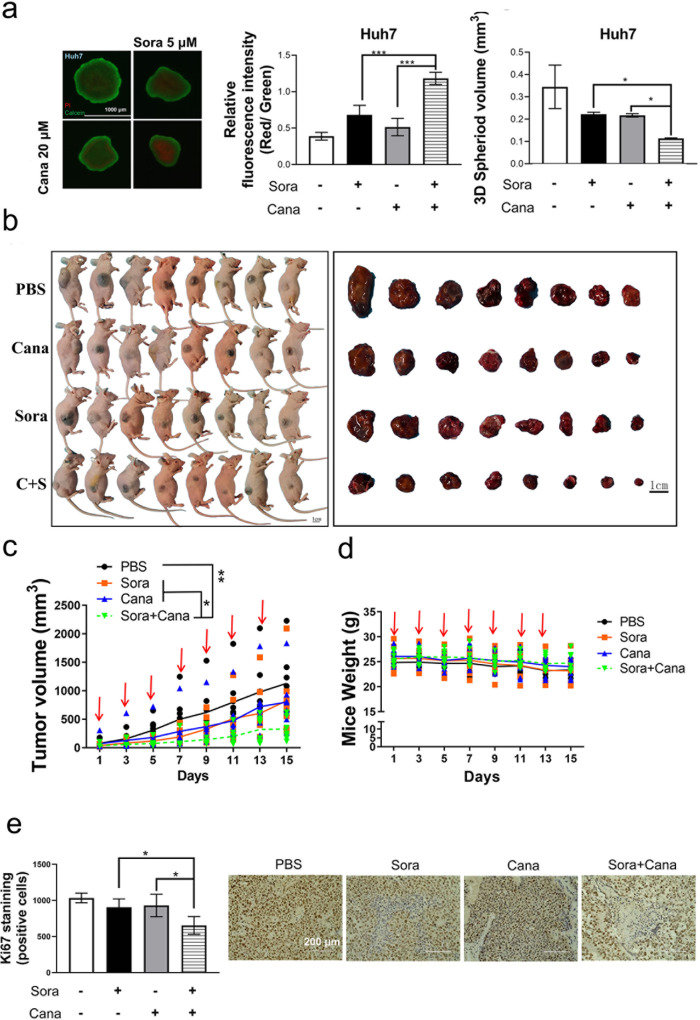

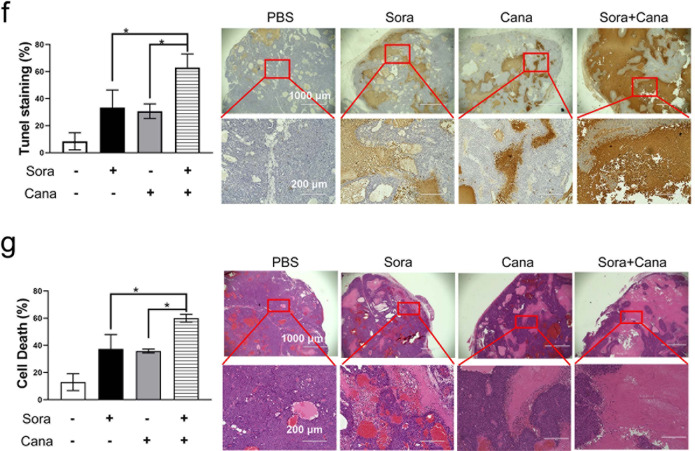
Combined treatment with canagliflozin and sorafenib inhibited HCC xenograft tumor growth and caused extensive cell death in vivo. **a** The 3D spheroid tumor models established with Huh7 cells were treated with 5 μM sorafenib, 20 μM canagliflozin or the combination for 72 h. The cell viability (middle panel) and volumetric quantification of spheroids (right panel) were measured. **b**, **c** Mice bearing inoculated Huh7 xenograft tumors were treated with sorafenib alone (20 mg/kg mouse weight), canagliflozin alone (30 mg/kg), the combination treatment (20 mg/kg sorafenib plus 30 mg/kg canagliflozin, C + S) or PBS for 15 days once every other day. The resected tumors were photographed (**b**), and the tumor sizes (**c**) were measured with a digital caliper. **d** After the indicated treatments, the mouse weights in the four groups were monitored every day. Summarized data are presented. **e** The proliferation status of xenograft tumor cells was determined by IHC with Ki-67 antibody. The Ki-67 staining results are summarized in the left panel. **f** Cell death was identified by TUNEL assay. **g** H&E pathological staining revealed that the combination treatment caused extensive cell death (as characterized by disrupted cell morphology and disappearance of stained nuclear). *P < 0.05.

Finally, we determined whether the combined treatment of canagliflozin and sorafenib was effective in inhibiting HCC xenograft tumor growth in vivo. The combination treatment, compared to canagliflozin alone or sorafenib alone, caused marked increase in cell death and a decrease in spheroid volume in both HepG2 and Huh7 3D spheroid models (Fig. 8a and Supplementary Fig. 5a–c). Then, the combination treatment was applied to HCC xenograft nude mice in vivo. Although both canagliflozin alone and sorafenib alone inhibited HCC xenograft tumor growth, the combined treatment almost terminated xenograft tumor growth (Fig. 8b, c). Moreover, IHC staining revealed that the combination treatment abolished Ki-67 staining (an indicator of cell proliferation, Fig. 8e, Supplementary Fig. 5d) and caused extensive cell death (Fig. 8f and Supplementary Fig. 5e, as determined by TUNEL staining). According to an H&E pathological examination (Fig. 8g and Supplementary Fig. 5f), after combination treatment, the xenograft tumor tissues exhibited larger regions of dead cells (~60%, as indicated by disrupted cell morphology and the disappearance of stained nuclei) than those treated with sorafenib alone (22%) or with canagliflozin alone (24%). Finally, the combination treatment was well tolerated in the tested mice, as indicated by a lack of change in either mouse weight (Fig. 8d) or behavior (data not shown) during the entire experimental period.

## Discussion

**Fig. 9:**
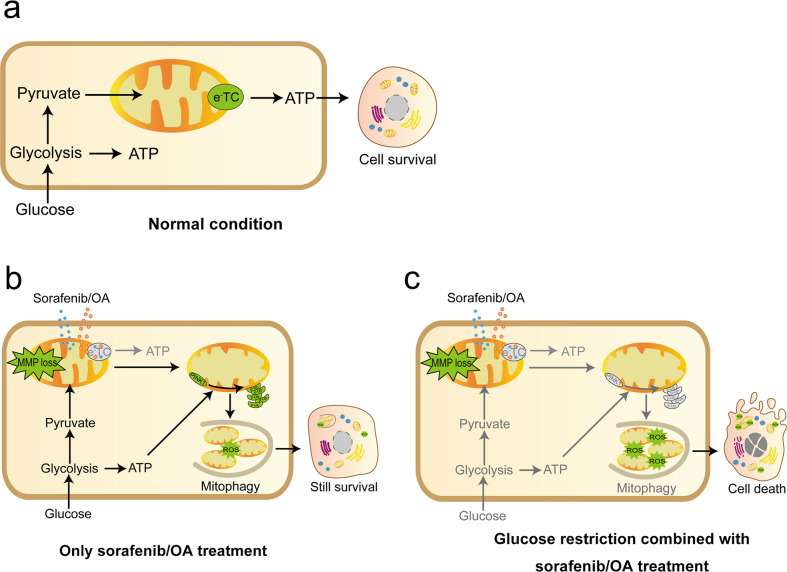
Graphic abstract. **a** Both the mitochondrial electron transport chain (eTC) and glycolysis supply energy in HCC cells. **b** Inhibition of eTC by sorafenib or oligomycin/antimycin (OA) impairs ATP generation. However, glycolysis maintains a low level of ATP generation to support mitophagy, which eliminates damaged mitochondria and prevents ROS accumulation. **c** The combination of sorafenib with glucose starvation (or with canagliflozin, a glucose transporter inhibitor) abolishes mitophagy and causes extensive cell death. This study may shed light on a potential application of transarterial sorafenib embolization (TASE).

This study demonstrated that simultaneous combination of sorafenib and glucose restriction (achieved by either forced glucose deprivation or the SLC5A2 inhibitor canagliflozin) synergistically induced HCC cell death. The combination treatments targeted two main energy metabolism pathways: mitochondrial respiration, targeted by the ETC poison sorafenib, and glycolysis, targeted by glucose restriction. The resultant ATP deletion sequentially abolished prosurvival mitophagy, exacerbated mitochondrial damage and ultimately caused cell death (Fig. 9).

To date, two Phase III (post-TACE and TACE-2) and two Phase II (SPACE and TACTICS) randomized controlled clinical trials have been performed to compared the combined efficacy of sequential treatments of sorafenib and TACE with TACE alone^10–13^. One main finding from these trials indicated that the combination treatment led to a better prognosis, particularly in time to progression (TTP), which was dependent on the duration of sorafenib treatment, suggesting an additive (not a synergistic) effect. In the post-TACE trial^10^, the subgroup patients from Korea who received sorafenib treatment for a relatively long period (a median of 31 weeks) presented a better TTP (hazard ratio [HR] = 0.38, 95% CI 0.18–0.81) than those who received TACE alone; however, a Japanese subgroup who received 16 weeks (median) of sorafenib treatment (HR = 0.94, 95% CI 0.75–1.19) failed to show an improved TTP. A similar trend was achieved in the SPACE trial^11^, in which an Asian subgroup receiving 30 weeks of sorafenib treatment (HR = 0.72, 95% CI 0.46–1.14, *P* = 0.078) was compared with a non-Asian subgroup who received 17.4 weeks of sorafenib treatment (HR = 0.87, 95% CI 0.58–1.30, *P* = 0.243). The TACE-2 trial was based on sorafenib administration for 17.1 weeks, and this therapy failed to promote progression-free survival (HR = 0.99, 95% CI 0.77–1.27)^12^. In contrast, the recent TACTICS trial based on the longest sorafenib treatment duration (median of 38.7 months) showed better progression-free survival (HR 0.59, 95% CI 0.41–0.87)^13^.

The synergistic effects were found to be dependent on the inhibition of both mitochondrial respiration and glycolysis. First, only sorafenib specifically synergized with glucose restriction (Fig. 1); neither antiangiogenic inhibitors (lenvatinib and brivanib) nor TACE chemotherapeutic drugs (cisplatin and doxorubicin) exerted effects similar to those of sorafenib (Fig. 2). This outcome is probably due to sorafenib, in contrast to the other tested drugs, directly represses ETC complex II/III activity^31^. In contrast, cisplatin and doxorubicin restored ATP levels after glucose restriction-induced ATP reduction (Fig. 2d). More importantly, cisplatin and doxorubicin protected against long-term glucose restriction-induced cell death (Fig. 2). In line with this outcome, cisplatin and doxorubicin have been shown to induce autophagy, which in turn renders osteosarcoma and other cancer cells resistant to treatment^40^. Clinical observations have been reported to show that the effect of TACE (with chemotherapeutic drugs) was not superior to TAE (without drugs) in terms of tumor responses and patient overall survival^3–5^. Thus, our findings may further dissuade the use of these drugs in TACE.

Second, the ETC poisons OA reproducibly synergized with glucose restriction to cause HCC cell death (Fig. 5), reinforcing the essential role played by mitochondrial respiration in HCC cell survival. Similar to sorafenib, OA alone triggered mitophagy, which was abolished when OA was combined with glucose restriction (Fig. 5). Direct administration of OA may also be useful for HCC treatment. Our recent study based on high-throughput chemical screening showed that oligomycin, as well as three other ETC inhibitors, significantly repressed SALL4-expressing HCC cell proliferation and patient-derived HCC xenograft tumor growth^41^. Notably, in the specific type of HCC examined in our previous study, the oncofetal protein SALL4 promoted mitochondrial respiration through transcriptional regulation. Furthermore, another recent study revealed that metformin synergized with hypoglycemia induced by fasting to impair xenograft tumor growth in mice^42^. Moreover, metformin (a first-line medication for type-II diabetes) exhibited an inhibitory effect on ETC complex I activity^43^. We also tested the combined effect of metformin and canagliflozin in a parallel xenograft study. The combination of these two antidiabetic drugs demonstrated a similar but moderate synergistic effect compared to that of sorafenib plus canagliflozin (data not shown). Our study thus supported the hypothesis that mitochondrial respiration and glycolysis are both essential for HCC cell survival.

Mitophagy is required for HCC cell survival. Upon mitochondrial injury, cells can activate mitophagy to eliminate mitochondria-enclosed proteins and redox species, which are toxic if released into cytosol^21,22^. Sorafenib or OA alone effectively caused dose- and time-dependent PINK1 accumulation and mitophagy (Figs. 4 and 5). PINK1 has been previously reported to be translationally regulated by glucose metabolism-mediated energy metabolism^44^; therefore, PINK1 abundance can be reduced by impaired ATP production. This mechanism may help explain how PINK1 was decreased when sorafenib treatment was combined with glucose restriction. With sorafenib or OA alone, cells accumulate PINK1 to induce mitophagy. However, when these drugs are combined with glucose deprivation, both ATP generation and PINK1 expression were completely inhibited. To determine whether PINK1 and mitophagy are dependent on glycolysis in other scenarios, further investigation is needed.

The marked increase in phospho-Ub chain modification, which is an “eat-me” signal, may trigger sorafenib-induced mitophagy, indicating the possible involvement of E3 ubiquitin ligases. In PRKN-independent mitophagy, several E3 ubiquitin ligases, such as SIAH1, MUL1, STUB1, and SMURF1, have been reported to regulate mitophagy^22^. In summary, these E3 ligases localize or translocate to the outer membrane surface of damaged mitochondria and add ubiquitin chains to proteins, triggering the recruitment of adapter proteins, including SQSTM1. The conjugation of MAP1LC3B initiates autophagosome formation and promotes subsequent lysosomal degradation. In our experiments, SIAH1, MUL1, and STUB1 were detectable in all tested HCC cell lines (Fig. 6a). However, only SIAH1 was found to be essential for phospho-Ub chain accumulation, mitophagy induction and cell survival (Fig. 6 and Supplementary Fig. 3). The essential role played by SIAH1 in mitophagy was first reported in SNCAIP-PINK1-triggered mitophagy^34^. In the brain, SNCAIP interacts with alpha-synuclein to form specialized structure presynaptic terminals in neuronal cells. SNCAIP recruits SIAH1 to depolarized mitochondria and enables the latter to ubiquitinate mitochondrial proteins^34^. However, whether SIAH1 plays a role in HCC carcinogenesis is still unknown.

The mitochondrion-residing protein MUL1 has been reported to compensate for the loss of PRKN in fruit flies and mouse cortical neurons^35^. MUL1 participates in gemcitabine-induced PINK1 stabilization and mitophagy induction in a PINK1-dependent but PRKN-independent manner^45^. In addition, STUB1 is a cytosolic E3 ligase. Its loss contributes to impaired mitophagy and swollen impaired mitochondria^36^. However, in our study, silencing either MUL1 or STUB1 failed to affect sorafenib-induced mitophagy (Supplementary Fig. 3). These results excluded their possible involvement in mitophagy in the treated HCC cells. With these two proteins used as negative controls, our study confirmed that SIAH1, at least partially, played an essential role in sorafenib-induced mitophagy. Because there are ~500–1000 E3 ubiquitin ligases in humans, other E3 ligases may act parallel to SIAH1; for example, ARIH1^46^ and HUWE1 may be involved^47^, warranting their future study.

By demonstrating that simultaneous treatments with sorafenib and glucose restriction synergistically induced HCC cell death, the results of the present study may have important clinical implications. Doxorubicin and/or cisplatin should be replaced with sorafenib in the intra-arterial infusion of TACE. First, this substitution may prevent unnecessary antagonizing effects of doxorubicin and cisplatin against starvation-induced cell death. Second, sorafenib-secreting beads can maximize its effect in the targeted region by synergizing with embolization-induced glucose restriction to achieve synthetic lethality. Third, direct administration (rather systemic treatment) of sorafenib into the embolization-affected region may prevent off-target effects in other sensitive tissues. Very recently, a study reported a sorafenib-secreting embolic microspheres used in combination with TACE for the treatment of a preclinical rat HCC model^48^. In the present xenograft study, we demonstrated that a combination treatment of sorafenib and canagliflozin (which inhibits the glucose transporter SLC5A2) significantly retarded HCC xenograft tumor growth. Systemic application of sorafenib and canagliflozin in clinical settings may affect other tissue regions with high SLC5A2 expression. Taken together, we suggest applying transarterial sorafenib embolization (TASE, with sorafenib-secreting beads) to achieve a synergistic effect in HCC treatment. This idea, however, needs to be extensively examined in future preclinical animal models and clinical trials.

In summary, the present study demonstrated that sorafenib specifically synergized with glucose restriction to induce HCC cell death in vitro and repress xenograft tumor growth in vivo. Mechanistically, sorafenib activated PINK1-SIAH1-mediated mitophagy. However, the combined treatment of sorafenib with glucose restriction (or canagliflozin treatment) affected both the ETC and glycolysis, two essential ATP-generating pathways. As a result, the combination treatment blocked mitophagy and caused cell death. The study may shed light on a direct combination of TAE with sorafenib (called TASE) in HCC treatment.

## Supplementary information

Supplementary data
