# Supplementary material for: Simultaneous treatment with sorafenib and glucose restriction inhibits hepatocellular carcinoma in vitro and in vivo by impairing SIAH1-mediated mitophagy
Source: Exp Mol Med. 2022 Nov 16;54(11):2007–21. doi: 10.1038/s12276-022-00878-x (PMC9723179; doi:10.1038/s12276-022-00878-x)
Supplement: Supplementary file 1 — Supplementary data [file 12276_2022_878_MOESM1_ESM.pdf]

Supplementary Material and Methods

Detection of reactive oxygen species (ROS) and mitochondrial membrane potential (MMP)

After indicated treatments, the cells were incubated with H_2DCFDA (DCF, for general ROS; #C6827) or mitoSOX-Red (for mitochondria derived ROS; #M36008); with tetramethylrhodamine methyl ester (TMRM; #I34361) and JC-1 (both for MMP; #M34152) from Thermo-Fisher. The changes of fluorescence intensities were measured using CytoFLEX FCM (Beckman, Atlanta, Georgia, USA) and then analyzed by FlowJo software.

IHC antibodies

In IHC experiemnts, expression of Ki-67 was determined using 1:300 diluted primary antibodies (#ab92742, Abcam) for 2 hours before standard biotin-streptavidin HRP detections and haematoxylin counterstaining (Zsbio, Beijing, China). The cell death in tumor tissues was determined by TUNEL assay (#C1090, Beyotime).

Measurement of glucose uptake

After Huh7 cells were seeded in 24-well plate overnight, the cells were pretreated with 1 μM insulin for 20 min to activate glucose transportation, and then treated with Canagliflozin (20 μM) or Phloretin (100 μM) for 1 hour, glucose uptake (2-DG-6-phosphate, 2-DG6P) of the cells were measured using a Glucose Uptake Colorimetric Assay Kit (#K676-100, Biovision).

siRNA reagents

The siRNA targeting PINK1 (HSS127945 and HSS185707), SIAH1 (HSS109741, HSS185553 and HSS185554), STUB1 (HSS145537, HSS145538 and HSS145539), and MUL1 (HSS128476, HSS128477 and HSS128478) were obtained from Thermo-Fisher.

Supplementary Fig. 1. Glucose restriction sensitized HCC cells to sorafenib-induced cell death *in vitro*.

a Meta-analysis of median time to progression (TTP) between the sorafenib plus TACE group and TACE alone group. **b-c** HepG2, Huh7 and HCCM cells were treated with sorafenib (10 μM) with or without glucose for 6hr. Cell death was determined by subG1 (**b**) and LDH release assay (**c**). *, P < 0.05; **, P < 0.01; and ***, P < 0.001.

Supplementary Fig. 2. Lenvatinib or brivanib failed to affect mitochondrial OCR.a Huh7 cells were treated with 10 μM lenvatinib or brivanib with or without glucose and then the mitochondrial OCRs were determined by the Seahorse metabolic assay as in **Fig. 3e**. **b** The relevant OCRs were calculated accordingly as in **Fig. 3f**. *P < 0.05; ***, P < 0.001.

Supplementary Fig. 3. MUL1 and STUB1 were not essential for sorafenib-induced mitophagy.

a siRNA-MUL1 Huh7 cells were treated with sorafenib (5 μM) for 20 hours and then the proteins were detected by Western blotting. **b** siRNA-STUB1 Huh7 cells were treated with sorafenib (5 μM) for 20 hours and then the proteins were detected by Western blotting.

Supplementary Fig. 4. SLC2A1 and SLC5A2 were over-expressed both in HCC clinical samples and in HCC cell lines.

a The expression difference and statistical significance for individual glucose transporter were shown in the table. **b** Heatmap of glucose transporter genes expression. N: nontumor liver tissues. T: HCC. **c** Both SLC2A1 (GLUT1) and SLC5A2 (SGLT2) were determined in multiple HCC cell lines by respective antibodies. **d** Huh7 cells were pretreated with 1 μM insulin to activate glucose transport, and then treated with Canagliflozin (20 μM) or Phloretin (100 μM) for 1 hour before measurement of glucose uptake.

Supplementary Fig. 5. The combined treatment of canagliflozin and sorafenib inhibited HCC xenograft.

a The 3D Spheroid tumor models of HepG2 were treated with 5 μM sora, 20 μM cana or the combination respectively for 48 hours. The cell viability (middle panel) and volumetric quantification of spheroid (right panel) were measured. **b-c** The 3D Spheroid tumor models

of Huh7 (**b**) HepG2 (**c**) were treated with 5 μM sora, 20 μM cana or the combination for 72 or 48 hours, respectively. **d-f** Images of Ki-67 (**d**), TUNEL (**e**) and H&E (**f**) staining of the xenograft nodules. The data were summarized in **Fig. 8e-g** left panel.

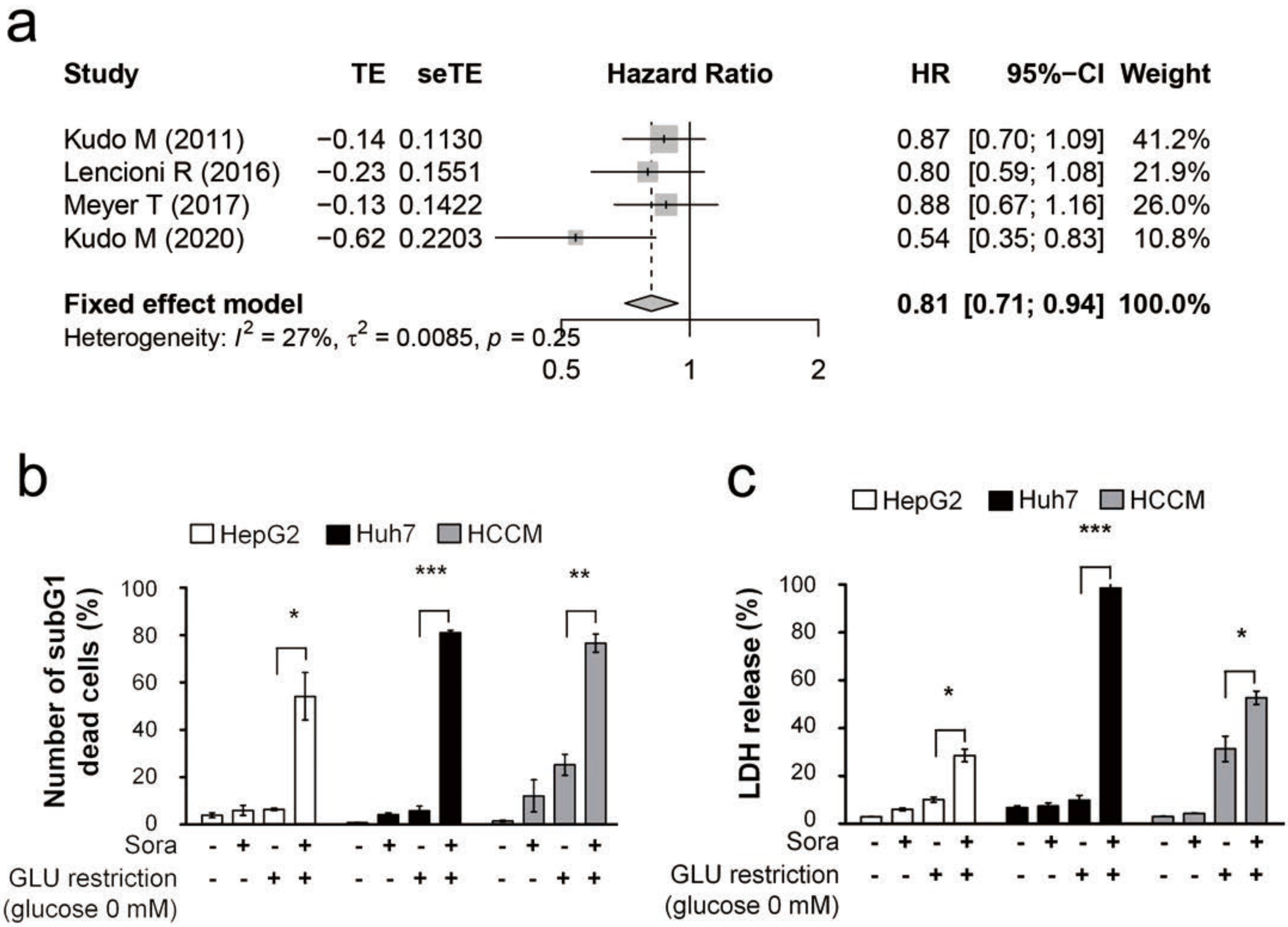
a
Study
TE
seTE
Hazard Ratio
HR
95%−CI
Weight
Kudo M (2011) −0.14 0.1130 0.87 [0.70; 1.09] 41.2%
Lencioni R (2016) −0.23 0.1551 0.80 [0.59; 1.08] 21.9%
Meyer T (2017) −0.13 0.1422 0.88 [0.67; 1.16] 26.0%
Kudo M (2020) −0.62 0.2203 0.54 [0.35; 0.83] 10.8%
Fixed effect model 0.81 [0.71; 0.94] 100.0%
Heterogeneity: I^2 = 27%, τ^2 = 0.0085, p = 0.25
0.5
1
2
b
HepG2
Huh7
HCCM
Number of subG1 dead cells (%)
100
80
60
40
20
0
*
***
**
Sora
GLU restriction (glucose 0 mM)
c
HepG2
Huh7
HCCM
LDH release (%)
100
80
60
40
20
0
*
***
*
Sora
GLU restriction (glucose 0 mM)

Figure S1

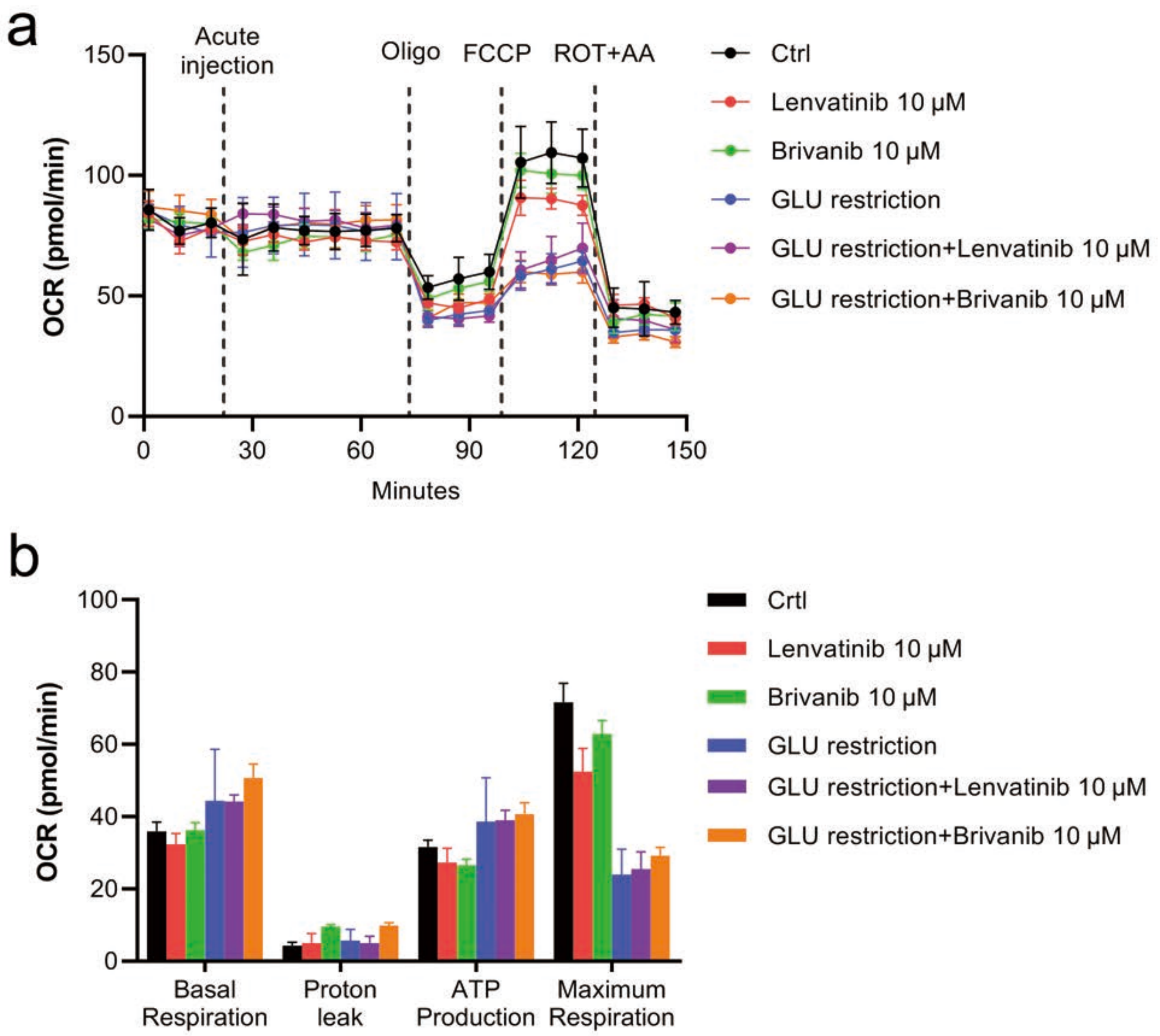
a
Acute injection
Oligo
FCCP
ROT+AA
OCR (pmol/min)
Minutes
Ctrl
Lenvatinib 10 μM
Brivanib 10 μM
GLU restriction
GLU restriction+Lenvatinib 10 μM
GLU restriction+Brivanib 10 μM
b
OCR (pmol/min)
Basal Respiration
Proton leak
ATP Production
Maximum Respiration
Crtl
Lenvatinib 10 μM
Brivanib 10 μM
GLU restriction
GLU restriction+Lenvatinib 10 μM
GLU restriction+Brivanib 10 μM

Figure S2

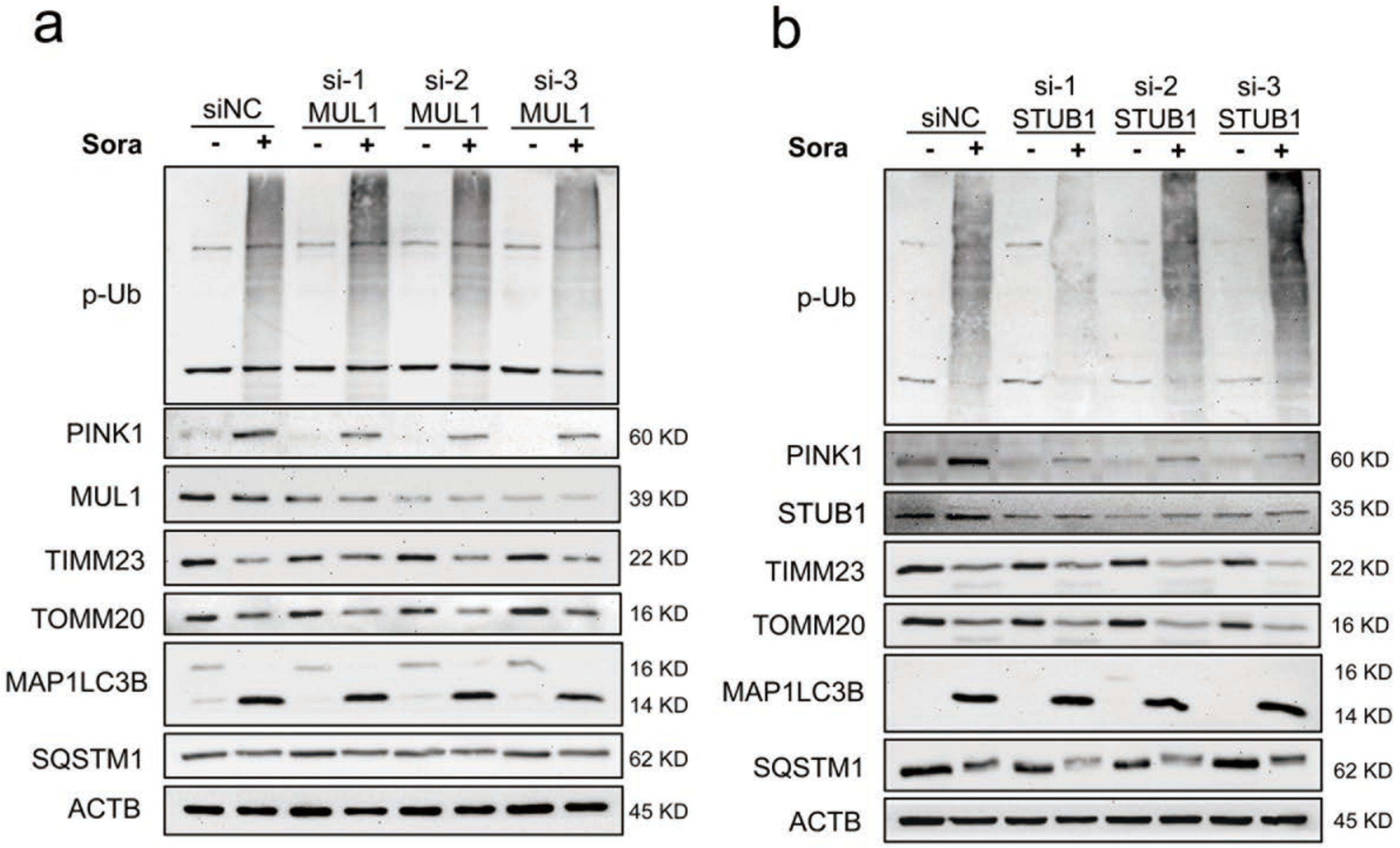
a
siNC
si-1 MUL1
si-2 MUL1
si-3 MUL1
Sora
- + - + - + - +
p-Ub
PINK1
60 KD
MUL1
39 KD
TIMM23
22 KD
TOMM20
16 KD
MAP1LC3B
16 KD
14 KD
SQSTM1
62 KD
ACTB
45 KD
b
siNC
si-1 STUB1
si-2 STUB1
si-3 STUB1
Sora
- + - + - + - +
p-Ub
PINK1
60 KD
STUB1
35 KD
TIMM23
22 KD
TOMM20
16 KD
MAP1LC3B
16 KD
14 KD
SQSTM1
62 KD
ACTB
45 KD

Figure S3

a

Gene	NT Mean	T Mean	LogFC	P_ Value
SLC2A1	0.68005	2.53400	1.89772	3.92E-05
SLC2A2	154.34460	104.97760	-0.55607	8.66E-09
SLC2A3	2.81209	2.30384	-0.28760	0.03071
SLC2A4	0.75918	1.54163	1.02193	0.00177
SLC5A1	0.92699	0.49717	-0.89881	5.91E-22
SLC5A2	0.02648	0.10491	1.98639	6.38E-10

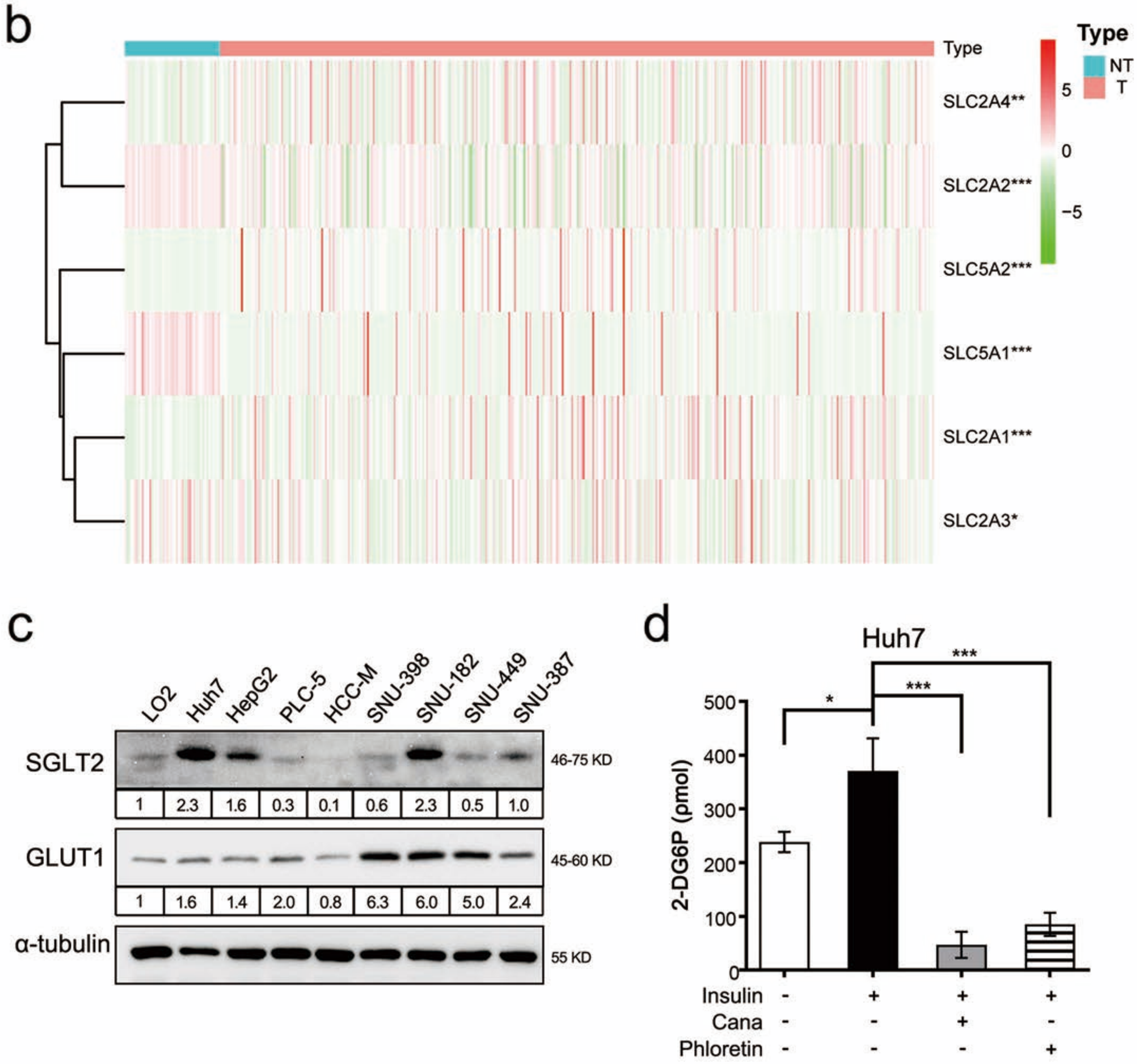

Figure S4

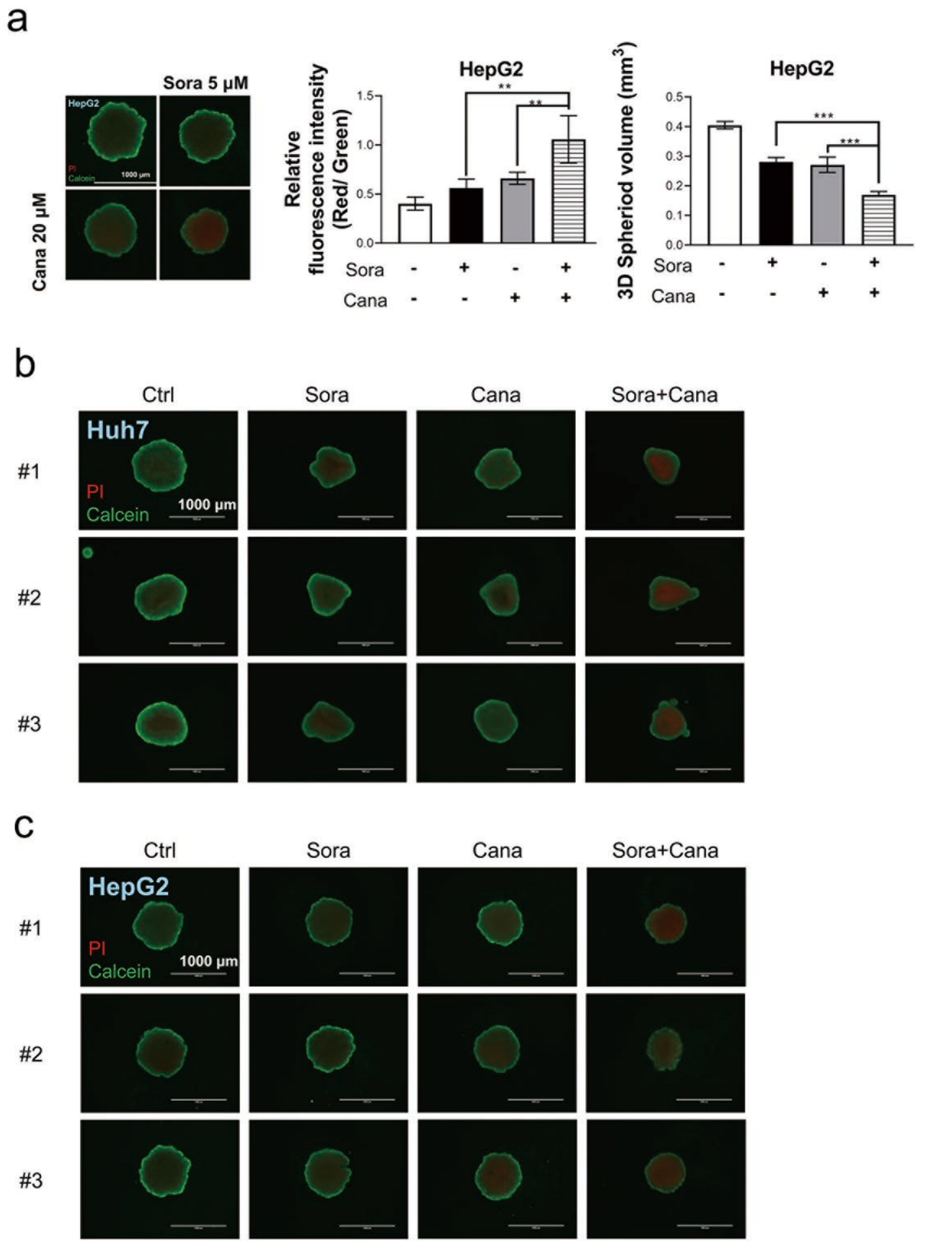
a
Sora 5 μM
Cana 20 μM
HepG2
PI
Calcein
1000 μm
HepG2
Relative fluorescence intensity (Red/ Green)
1.5
1.0
0.5
0.0
**
**
Sora - + - +
Cana - - + +
HepG2
3D Spheriod volume (mm3)
0.5
0.4
0.3
0.2
0.1
0.0
***
***
Sora - + - +
Cana - - + +
b
Ctrl
Sora
Cana
Sora+Cana
Huh7
PI
Calcein
1000 μm
#1
#2
#3
c
Ctrl
Sora
Cana
Sora+Cana
HepG2
PI
Calcein
1000 μm
#1
#2
#3

Figure S5(a-c)

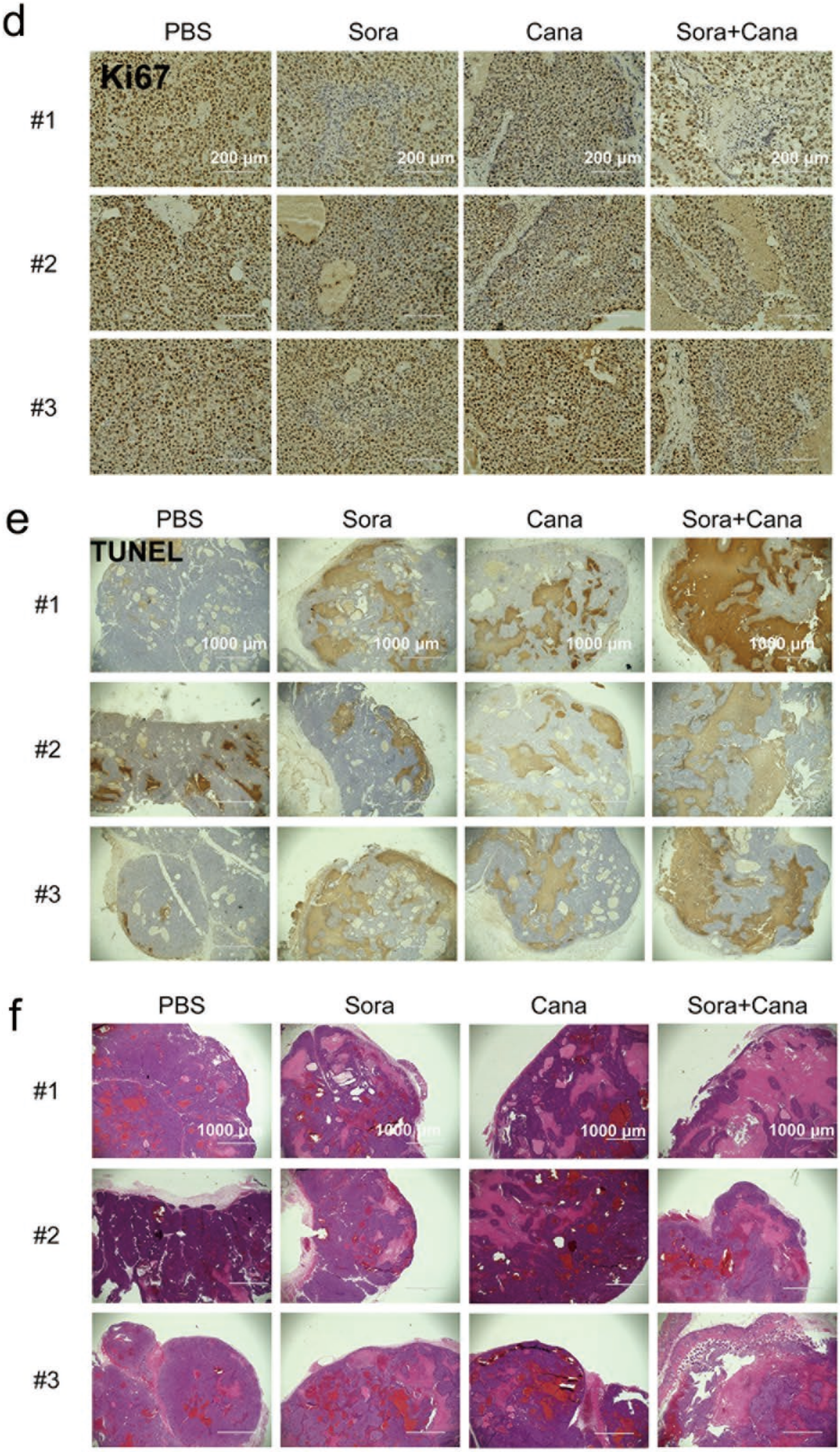

Figure S5(d-f)
